# Prognostic Significance of Ki67 Expression in Prostate Cancer in Nigerians: A Single-Center Study

**DOI:** 10.7759/cureus.80997

**Published:** 2025-03-22

**Authors:** Bamnan Dallang, Kevin N Ezike, Innocent Emmanuel, Barnabas M Mandong, Ayuba M Dauda, Philip O Akpa, Emmanuel E Oguntebi

**Affiliations:** 1 Anatomic Pathology and Forensic Medicine, Nile University of Nigeria, Abuja, NGA; 2 Anatomic Pathology and Forensic Medicine, Jos University Teaching Hospital, Jos, NGA; 3 Pathology, University of Jos, Jos, NGA; 4 Anatomic Pathology and Forensic Medicine, Asokoro District Hospital, Abuja, NGA

**Keywords:** gleason score, immunohistochemistry, ki67 proliferative index, nigeria, prostate cancer

## Abstract

Introduction

Prostate cancer is one of the most common cancers occurring in men and one of the leading causes of cancer deaths globally. Associated risk factors include age, race, and positive family history. Older patients have an increased risk for more aggressive forms. Its incidence is particularly high in Black men. The Gleason grading and scoring system is an established prognostic factor for prostate cancer. Ki67, a nuclear protein, coded for by the MKi67 gene located on chromosome 10q26.2 and detected in all phases of the cell cycle, provides information on the proliferation index of cancer cells, including prostate cancer. This study is purposed to establish the significance of Ki67 expression as a prognostic marker of prostate cancer by correlating it with patients’ age and Gleason scores, respectively, with the aim of understanding the molecular characteristics of prostate cancers in our environment, in order to assist in categorizing patients for treatment.

Materials and methods

This was a retrospective study carried out in Jos University Teaching Hospital (JUTH), Jos, Nigeria, involving histologically diagnosed prostate cancers over a five-year period. The surgical pathology reports and information on patients’ biodata and clinical features were retrieved from departmental records and the hospital’s electronic records. The appropriate archival hematoxylin and eosin (H&E)-stained slides and their respective formalin-fixed paraffin-embedded (FFPE) tissue blocks were retrieved and reviewed, with new sections made when necessary. Immunohistochemical analysis to assess the Ki67 proliferative index was carried out on sections made from representative blocks of each case using Ki67 monoclonal antibodies, according to the established protocol prescribed by the manufacturers. The Ki67 proliferative index was categorized as negative, low, and high. Data obtained were analyzed, and results were presented as percentages/frequencies and displayed as tables and charts.

Results

One hundred forty-two cases met the inclusion criteria. The age range was 30-90 years. The peak age group was 70-79 years with 38% (54/142). Majority, 81.7% (117/142), of cases occurred in patients above 60 years old. An overwhelming majority, 71.8% (102/142), of cases were poorly differentiated adenocarcinomas (Gleason scores 8-10), 21.1% (30/142) were moderately differentiated (Gleason score 7), and 7.0% (10/142) were well-differentiated (Gleason score 6). Among high Ki67 proliferative index cases, 81.4% (57/70) were aged above 60 years. Similarly, 85.1% (40/47) of low proliferative index cases were also aged above 60 years. Additionally, 92.5% (37/40) of well- and moderately differentiated cancers (Gleason scores 6 and 7) had negative or low Ki67 proliferative indices, while 65.7% (67/102) of the poorly differentiated (Gleason scores 8-10) had high indices.

Conclusion

Our study demonstrated a direct correlation of the Ki67 proliferative index with both histologic grade and aggressiveness of prostate carcinoma in a Nigerian population thereby confirming high Ki67 proliferative index as an adverse prognostic factor in prostate cancer. It, however, showed no direct relationship between age and Ki67 proliferative index. Determination of the Ki67 proliferative index is recommended for routine assessment of prostate cancer patients to help in risk stratification and instituting treatment plans.

## Introduction

Globally, prostate cancer is the second most common and also the second most frequently diagnosed cancer in men, after lung cancer [1,2]. Prostate cancer incidence increases with age, with only 1% of these cancers being clinically detected in men less than 50 years of age [1]. There are marked disparities in incidence between different geographical regions, being highest in North America with an estimate of 83.2-173.7 per 100,000 [3]. Racial discrepancies have been observed in relation to the incidence and mortality rates, with the highest rates occurring in Blacks [3]. Even in highly prevalent regions such as the USA, the incidence and mortality rate of prostate carcinoma in African American men is 2.4 times that of Caucasian American men [3].

The WHO classification of tumors of the prostate recognizes the following categories: epithelial, neuroendocrine, mesenchymal, hematolymphoid, metastatic, miscellaneous, and tumors of the seminal vesicles [1]. Malignant epithelial tumors predominate, and of these, adenocarcinomas occur most frequently [1,4].

The etiology of prostate cancer is not fully understood; however, risk factors include increasing age, race, family history, hormone levels, and other environmental factors [1,4]. The pathogenesis of prostate cancer is linked to the interaction between several genetic and environmental factors, with androgens playing a very vital role (just as they are important for the growth and survival of normal prostate tissue) as evidenced by tumor regression/atrophy following castration or other androgen ablation therapies [4].

The diagnosis of prostate cancer is usually confirmed by histological examination of prostate biopsy specimens, and it is graded using the Gleason scoring system [5,6]. The International Society of Urological Pathology (ISUP) has recently adopted prognostic grade groups based on the Gleason system, and these grade groups have a huge clinicopathological impact [5].

The Ki67 protein is a nuclear protein, coded for by the MKi67 gene located on chromosome 10q26.2 [6]. The Ki67 antigen is not detected in nuclei of cells that are out of the cell cycle (Go phase); however, in cells that are in the cell cycle, it can be detected in all phases (G1, S, G2, and M) with its expression markedly increased in the S phase, making it an excellent marker of cellular proliferation in immunohistochemistry [6]. Ki67 is one of the most widely studied molecular biomarkers in prostate cancers with several studies showing it to be of prognostic value, both independently and in conjunction with other prognostic indicators, especially the Gleason grades of these cancers [7-9].

This study is purposed to establish the significance of Ki67 expression as a prognostic marker of prostate cancer in our environment, to fill a gap arising from a dearth of studies on this subject, in order to identify and better treat patients whose cancers have an increased potential for aggressive behavior due to a high Ki67 proliferative index, regardless of the histologic grade. Ki67 expression will also be correlated with patients’ age and histologic grade, respectively, to assess its relationship, if any, with these variables. Finally, we will compare our findings with similar studies both within and outside Nigeria.

## Materials and methods

Study location and design

This was a retrospective study carried out in Jos University Teaching Hospital (JUTH), Jos, Nigeria, involving histologically diagnosed prostate cancers over a five-year period, specifically from January 1, 2015, to December 31, 2019. JUTH is a tertiary healthcare center with an active urology unit and anatomic pathology and forensic medicine department.

The surgical pathology reports of all trucut biopsy cases diagnosed as prostate cancer within the study period were retrieved from departmental records, while information on patients’ biodata and clinical features was obtained from the hospital’s electronic records. The appropriate archival hematoxylin and eosin (H&E)-stained slides and their respective formalin-fixed paraffin-embedded (FFPE) tissue blocks were retrieved. The slides were reviewed, and new sections were made from the FFPE tissue blocks of those with poor or deteriorated quality. In cases where the slides were missing but FFPE tissue blocks were found, new sections were also made. All the new sections were stained with H&E and reviewed. All the slides thus obtained for each case were subsequently reviewed by four pathologists in the study to confirm by consensus the diagnoses and other relevant morphological features and select those suitable for immunohistochemistry. In the event that the slides’ review yielded a different diagnosis, the protocol for informing the managing clinician and patient, as per extant JUTH Institutional Research Ethical Committee guidelines, was followed. Immunohistochemical analysis was carried out on sections taken from representative blocks of each case using Ki67 monoclonal antibodies. The Ventana (Roche Diagnostics, Indianapolis, IN, US) antibody and detection kit was utilized. All the samples were processed according to the established protocol prescribed by the manufacturers of the antibodies.

Inclusion and exclusion criteria

The inclusion criteria include all trucut biopsy cases diagnosed as prostate cancer within the study period in which the H&E-stained slides and FFPE tissue blocks were retrieved and cases for which the H&E-stained slides were missing but FFPE tissue blocks were found. The exclusion criteria include cases with missing or incomplete biodata and clinical features and cases in which the tissue blocks could not be retrieved, the FFPE tissue blocks had insufficient tissue to permit further sectioning for immunohistochemistry, and the diagnosis changed after review from malignant to equivocal, inconclusive, or benign.

Diagnostic criteria

The cases were classified according to the 2016 WHO classification of prostate tumors [1]. They were categorized based on the Gleason score as follows: well-differentiated (6), moderately differentiated (7), and poorly differentiated (8-10) [5].

The Ki67 proliferative index was categorized based on the percentage score as follows: negative (≤2%), low (>2%-30%), and high (>30%). To obtain the percentage score, all cells with nuclear staining for the Ki67 monoclonal antibody were counted as positive; the Ki67 labeling index was then calculated by the ratio of the number of positive cells to 200 tumor cells counted (in the most intensely staining areas) and expressed as percentages [10-14].

Statistical analysis

Data obtained were entered in a spreadsheet of Microsoft Office 365 version of the Excel software program (Microsoft Corporation, Redmond, WA, US) and analyzed with the Statistical Package for the Social Sciences (SPSS) Statistics for Windows, version 23.0 (IBM Corporation, Armonk, NY, US). Continuous variables were summarized using descriptive statistics, including range, median, and mean ± standard deviation, while categorical variables were presented as frequencies and percentages. The data was presented in the form of tables and charts. Inferential statistics (chi-squared test statistic) were used to find the association between two categorical variables. A p-value <0.05 was considered statistically significant.

Ethical consideration

Ethical clearance was obtained from the JUTH Institutional Research Ethical Committee via approval number JUTH/DCS/IREC/127/XXXI/2344.

## Results

**Figure 1:**
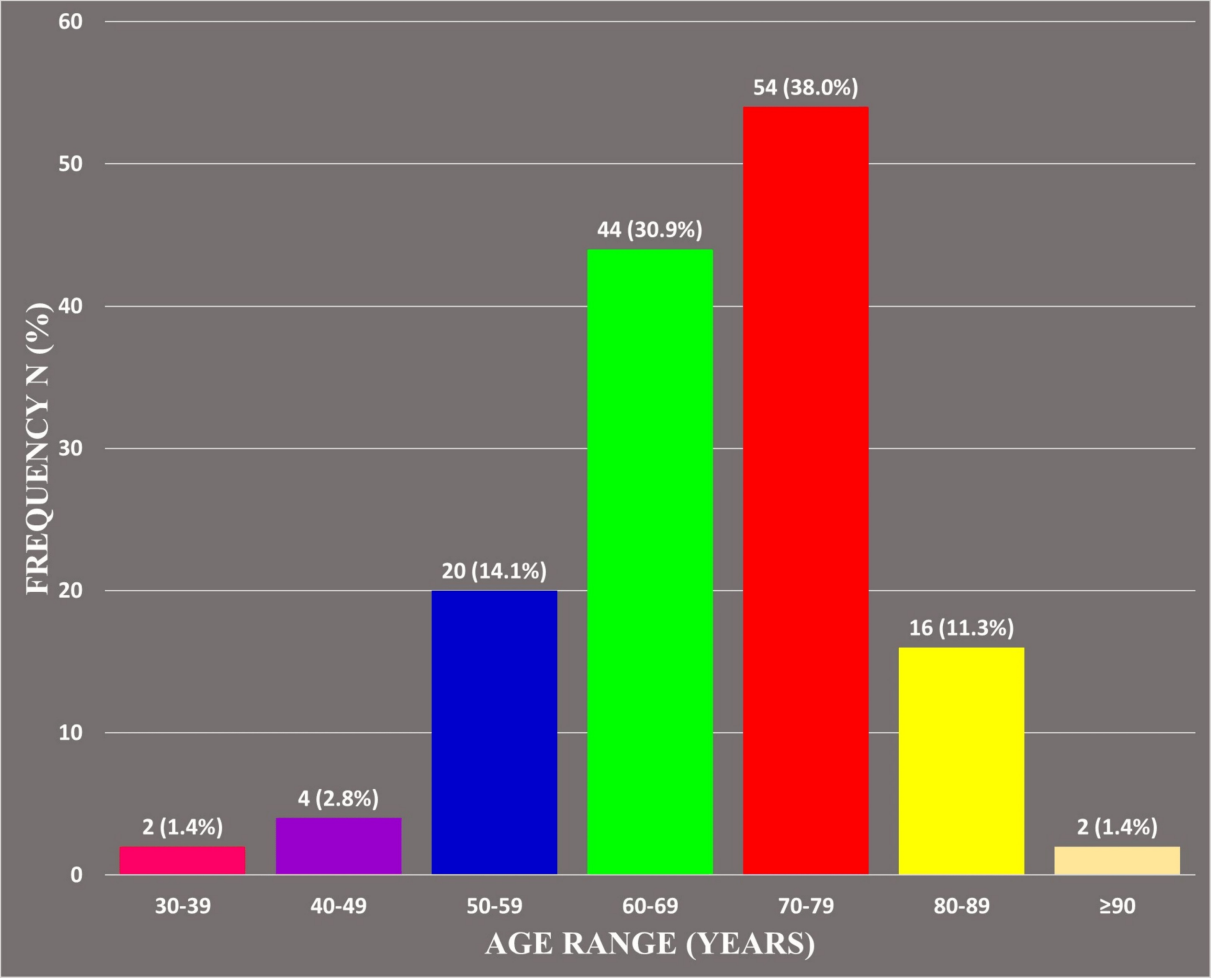
Age Distribution of Prostate Cancers

One hundred forty-two cases met the inclusion criteria. The age range was 30-90 years. The mean age was 67.7 ± 15 years while the median age was 70 years. The peak age range was 70-79 years with 38% (54/142). Majority of cases, 81.7% (117/142), occurred in patients above 60 years old (Figure 1).

**Figure 2:**
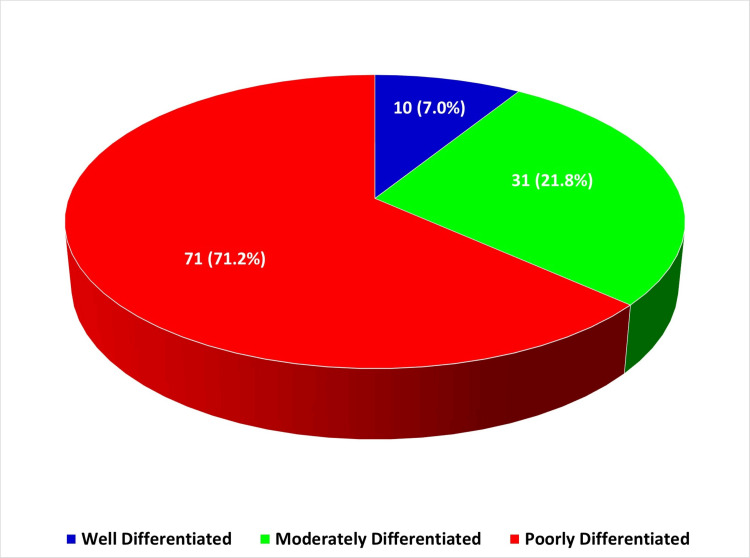
Distribution of Histological Grading of Prostate Adenocarcinoma

All the cases of prostate cancer seen within the study period were adenocarcinomas. These adenocarcinomas were categorized, based on their Gleason scores, into well-differentiated (6), moderately differentiated (7), and poorly differentiated (8-10). An overwhelming majority, 71.8% (101/142), were poorly differentiated (Figure 2).

**Figure 3:**
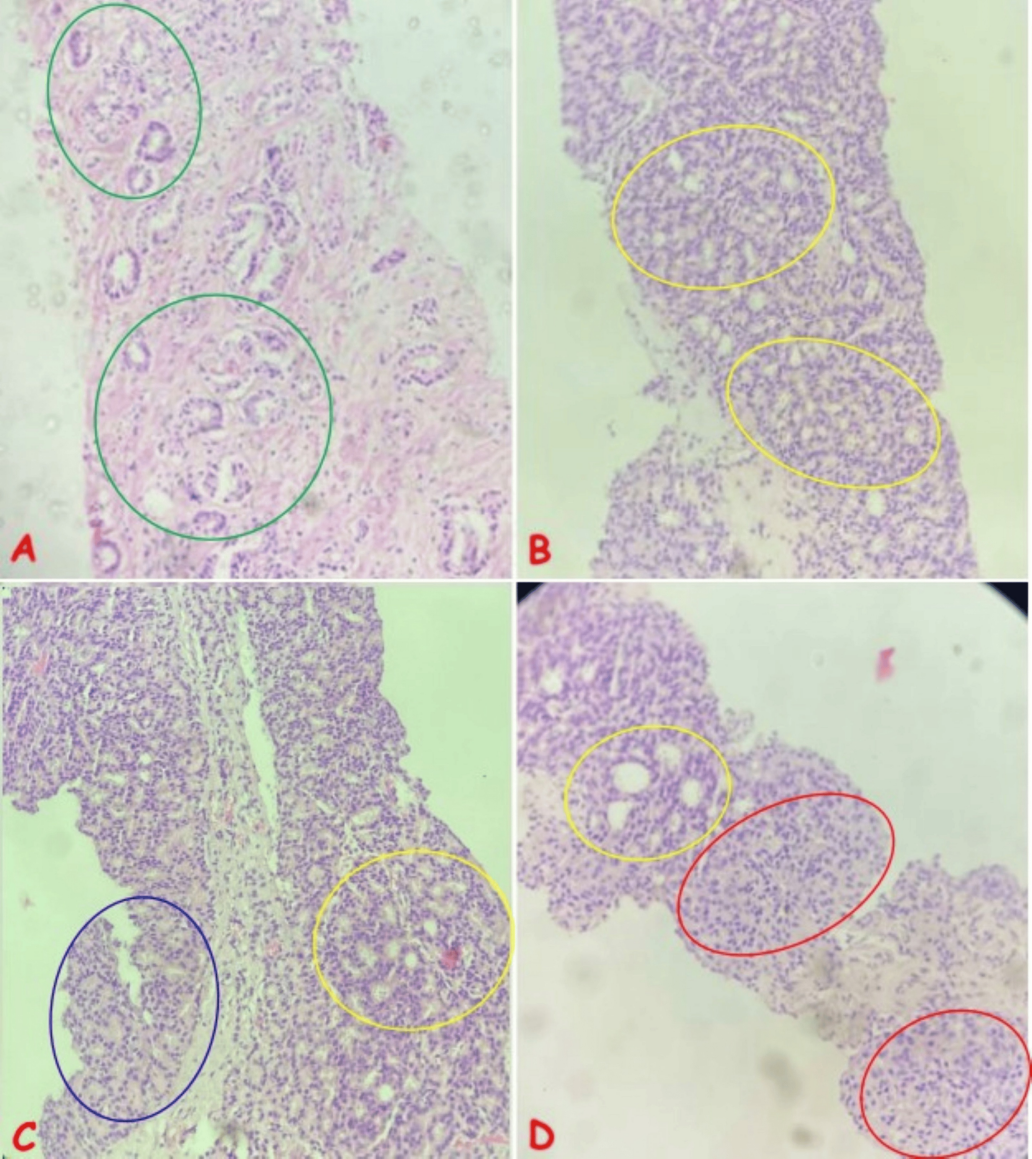
Photomicrographs Showing the Histological Features of Different Gleason Scores of Prostatic Adenocarcinoma (A) Adenocarcinoma, Gleason score 3+3 in a 65-year-old man with symptoms of bladder outlet obstruction; note the well-formed, variably sized atypical acini lined by a layer of dysplastic epithelium (green circles), invading the fibromuscular stroma (H&E, x200). (B) Adenocarcinoma, Gleason score 4+4 in a 58-year-old man with hematuria and lower back pain; note the irregular cribriform acini with small round lumina (yellow circles) occurring in closely packed nodular aggregates (H&E, x200). (C) Adenocarcinoma, Gleason score 4+4 in a 62-year-old man with lower urinary tract symptoms; note two different patterns of Gleason grade 4: irregular cribriform acini with small round lumina (yellow circle) and poorly formed atypical acini lacking lumina and fusing together (blue circle) separated by fibromuscular stroma (H&E, x200). (D) Adenocarcinoma, Gleason score 5+4 in a 58-year-old man with lower back pain and elevated serum PSA; note sheets of atypical cells (red circles) and large irregular cribriform glands with well-formed lumina (yellow circle) merging into each other (H&E, x100). H&E: hematoxylin and eosin; PSA: prostate-specific antigen

Within the poorly differentiated category, Gleason score 9 occurred most frequently, accounting for 42.6% (43/101) of cases, with Gleason grade combinations of 28/43 (65.1%) for 4+5 and 15/43 (34.9%) for 5+4, respectively. For the moderately differentiated cases (Gleason score 7), the Gleason grade combinations were 10/31 (32.3%) for 3+4 and 21/31 (67.7%) for 4+3, respectively (Table 1 and Figure 3).

**Table 1:** Histological Grading of Prostate Cancer by Gleason Score and ISUP Grade Group ISUP: International Society of Urological Pathology

Gleason grades	Gleason score	ISUP grade group	Frequency N (%)		Histologic grade
			Individual	Cumulative	
3+3	6	1	10 (7.0%)	10 (7.0%)	Well-differentiated
3+4	7	2	10 (7.0%)	31 (21.8%)	Moderately differentiated
4+3	7	3	21 (14.8%)		
3+5	8	4	7 (4.9%)	101 (71.2%)	Poorly differentiated
4+4	8	4	20 (14.1%)		
5+3	8	4	13 (9.2%)		
4+5	9	5	28 (19.7%)		
5+4	9	5	15 (10.6%)		
5+5	10	5	18 (12.7%)		
Total			142 (100%)	142 (100%)	

The relationship between age and the Ki67 proliferative index was not statistically significant. A high Ki67 proliferative index was seen in 49.1% (57/116) of cases in patients above 60 years, while in patients aged below 60 years, it was 50.0% (13/26). The two patients aged 90 years and above exhibited a high Ki67 proliferative index. The majority, 81.4% (57/70), of the cases with a high Ki67 proliferative index were in patients aged above 60 years. Similarly, 85.1% (40/47) of low proliferative index cases were also aged above 60 years (Table 2).

**Table 2:** Association of Age and Ki67 Proliferative Index of Prostate Adenocarcinomas Ki67 proliferative index: negative: ≤2%; low: >2%-30%; high: >30% χ^2^: chi-squared statistic

Variable	Ki67 proliferative index N (%)				χ^2^	p-value
	Negative	Low	High	Total		
Age					9.349	0.673
30-39	1 (0.7%)	0 (0.0%)	1 (0.7%)	2 (1.4%)		
40-49	1 (0.7%)	1 (0.7%)	2 (1.4%)	4 (2.8%)		
50-59	4 (2.8%)	6 (4.2%)	10 (7.0%)	20 (14.1%)		
60-69	10 (7.0%)	16 (11.3%)	18 (12.7%)	44 (31.0%)		
70-79	7 (4.9%)	21 (14.8%)	26 (18.3%)	54 (38.0%)		
80-89	2 (1.4%)	3 (2.1%)	11 (7.7%)	16 (11.3%)		
≥90	0 (0.0%)	0 (0.0%)	2 (1.4%)	2 (1.4%)		
Total	25 (17.6%)	47 (33.1%)	70 (49.3%)	142 (100%)		

**Figure 4:**
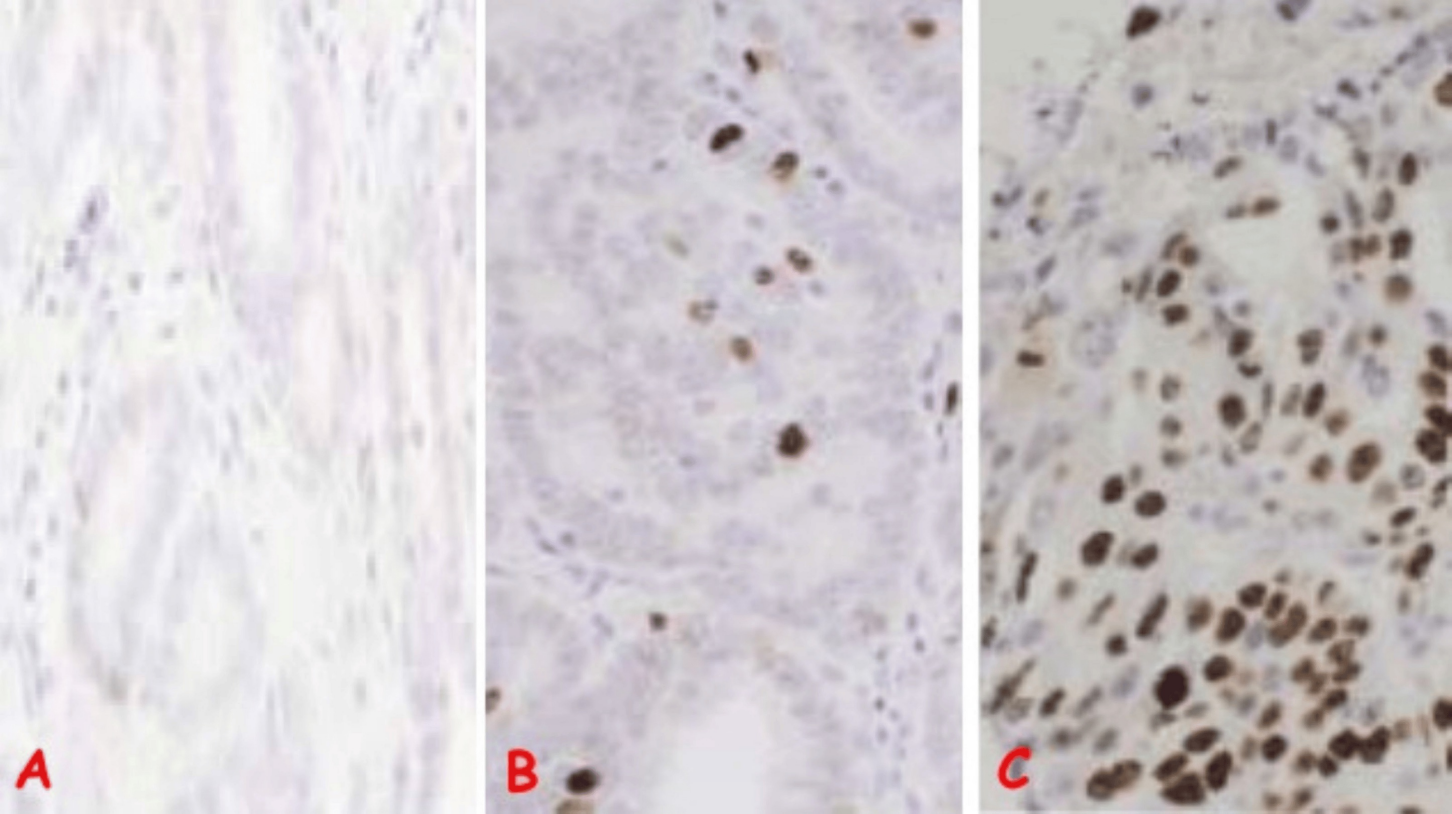
Photomicrographs of Immunohistochemistry for Various Ki67 Proliferative Indices (A) Negative Ki67 proliferative index in a case of adenocarcinoma, Gleason score 3+3 in a 63-year-old man with lower urinary tract symptoms; note the lack of nuclear staining of lesional cells. (B) Low Ki67 proliferative index in a case of adenocarcinoma, Gleason score 3+3 in a 60-year-old man with lower urinary tract symptoms; note <5% nuclear staining (dark brown coloration) of lesional cells (Ki67 IHC, x400). (C) High Ki67 proliferative index in a case of adenocarcinoma, Gleason score 4+5 in a 63-year-old man with lower urinary tract symptoms and lower back pain; note >30% nuclear staining (dark brown coloration) of lesional cells (Ki67 IHC, x400). IHC: immunohistochemistry

Overall, there were 82.4% (117/142) cases that had either a low or high Ki67 proliferation index, while only 17.6% (25/142) cases were negative proliferation markers. The relationship between the histologic grade and the Ki67 proliferative index was statistically significant. The majority, 92.5% (37/40), of well- and moderately differentiated adenocarcinomas (Gleason scores 6 and 7) had negative or low Ki67 proliferative indices, while in the majority, 65.3% (66/101), of the poorly differentiated (Gleason scores 8-10), the indices were high. Among the cases with Gleason score 9, both the 4+5 and 5+4 combinations had significantly more high Ki67 proliferative index than low, with 4+5 having 82.1% (23/28) high index and 17.9% (5/28) low index and 5+4 having 93.3% (14/15) high index and 0.7% (1/15) low index. In contrast, among the cases with Gleason score 7, the 3+4 combination had more cases with a negative index, 70% (7/10), than with low and high indices, 30% (3/10), while the 4+3 combination had more low and high index cases, 85.7% (18/21), than negative index cases, 14.3% (3/21) (Table 3 and Figure 4).

**Table 3:** Association of Gleason Score and Ki67 Proliferative Index of Prostate Adenocarcinomas Ki67 proliferative index: negative: ≤ 2%; low: >2%-30%; high: >30% ISUP: International Society of Urological Pathology; χ^2^: chi-squared statistic *Significant at 95%

Variables	Gleason grades	Gleason score	ISUP grade group	Ki67 proliferative index N (%)				χ^2^	p-value
				Negative	Low	High	Total		
Histologic grade								44.125	<0.001*
Well-differentiated	3+3	6	1	6 (4.2%)	3 (2.1%)	1 (0.7%)	10 (7.0%)		
Moderately differentiated	3+4	7	2	7 (4.9%)	2 (1.4%)	1 (0.7%)	31 (21.8%)		
	4+3	7	3	3 (2.1%)	16 (11.3%)	2 (1.4%)			
Poorly differentiated	3+5	8	4	6 (4.2%)	1 (0.7%)	0 (0.0%)	101 (71.2%)		
	4+4	8	4	0 (0.0%)	17 (12.0%)	3 (2.1%)			
	5+3	8	4	1 (0.7%)	1 (0.7%)	11 (7.7%)			
	4+5	9	5	0 (0.0%)	5 (3.5%)	23 (16.2%)			
	5+4	9	5	0 (0.0%)	1 (0.7%)	14 (9.9%)			
	5+5	10	5	2 (1.4%)	1 (0.7%)	15 (10.6%)			
Total				25 (17.6%)	47 (33.1%)	70 (49.3%)	142 (100%)		

## Discussion

Racial disparities in prostate cancer incidence are well documented, but its age incidence does not exhibit racial disparities, occurring across all racial groups over a wide age range, but by far more commonly in older males, peaking in the seventh and eighth decades [1,3,15-18]. Similarly, our study showed a preponderance of cases in the seventh and eighth decades with the eighth decade being the peak age range. Apart from the fact that cancers generally occur more in older persons due to the accumulation of genetic damage, the specific reasons for prostate cancer occurring more in older men despite the fact that serum levels of testosterone and insulin-like growth factor 1 (IGF-1), both of which are associated with its pathogenesis decline with age, are unclear [19]. It has been postulated that since the prostate gland continues to increase in size with age, there is a commensurate increase in the likelihood of malignant transformation [19]. It has also been suggested that with increasing life expectancy, more men live long enough to eventually develop prostate cancer and other cancers [19]. Furthermore, in some susceptible persons, with age, the prostate exhibits increased sensitivity to even small amounts of testosterone [20,21].

The Gleason scoring system has been shown to correlate with the prognosis of prostate cancer [4,6]. It has been evolving with successive reviews by the ISUP, and one of the major effects of the more recent grading systems has been a shift toward higher grades being reported in biopsies, which is the case in this study [5,21]. Other factors that may have contributed to high-histologic-grade carcinomas in our study include the lack of a proper screening program and late presentation [22,23]. This finding of the preponderance of high-histologic-grade prostate carcinomas in our study is consistent with findings in other Nigerian studies and studies from elsewhere involving other racial groups, suggesting that there is no racial disparity in the histologic grade [12,16,24,25].

The Ki67 protein is expressed in all cell cycle phases and Is known to be responsible for localizing nucleolar material to the mitotic chromosome periphery and for structuring perinucleolar heterochromatin [26]. However, it is not essential in normal cell proliferation because recent studies have demonstrated that loss of Ki67 has little or no impact on cell proliferation [26]. Nevertheless, its role in carcinogenesis and consequently as a marker of cell proliferation is well established [6,26]. Studies have shown that there is no direct correlation between age and Ki67 proliferative index in prostate cancer, and this trend is corroborated by the findings in this study [14,27]. This lack of correlation between age and Ki67 proliferative index may appear paradoxical given that it is widely established and accepted that there is a direct correlation between increasing age and higher histologic grade [28]. The explanation for this is that although the Ki67 proliferative index directly correlates with histologic grade, it is more accurate as a predictor of tumor aggression [29]. Our study findings are consistent with these two aspects of the relationship between the Ki67 proliferative index and histologic grade and tumor aggression. On the one hand, 65.3% of poorly differentiated adenocarcinomas had high Ki67 proliferative indices, while 92.5% of well- and moderately differentiated adenocarcinomas had negative or low indices, signifying a direct correlation of Ki67 proliferative index with histologic grade. On the other hand, among the cases with Gleason score 7, 70% of the 3+4 combination had negative indices, while 85.7% of the 4+3 combination had either low or high indices, signifying the correlation of the Ki67 proliferative index.

Limitations

The major limitation of this study was that it was hospital-based and, therefore, analyzed only the prostate biopsies received at our center. Accordingly, the findings may not be wholly representative of the prognostic significance of the Ki67 proliferative index in prostate cancer in the general population. Another limitation was the retrospective nature of the study design, which was associated with incomplete data and lack of complete retrieval of FFPE tissue blocks, both of which may have introduced distortions to the statistics. Inadequate storage and, hence, possible deterioration of archival FFPE tissue blocks may have influenced outcomes of the Ki67 proliferative index immunohistochemical assay especially the high numbers of negative indices in moderate- and high-histologic-grade carcinoma cases.

## Conclusions

Our study demonstrated that the Ki67 proliferative index not only correlates positively with poorly differentiated cancers but is also a predictor of aggressive potential in well- and moderately differentiated cancers in a Nigerian population because it can also be high in a subset of well- and moderately differentiated cancers. It, however, showed no direct relationship between age and the Ki67 proliferative index. More extensive and nationwide studies are required to confirm these findings. Assessment of the Ki67 proliferative index is, therefore, recommended in the routine assessment of prostate cancer patients to help in risk stratification and planning of treatment modalities.

## References

[1] Humphrey PA, Moch H, Cubilla AL, Ulbright TM, Reuter VE (2016). The 2016 WHO classification of tumours of the urinary system and male genital organs-part B: prostate and bladder tumours. Eur Urol.

[2] Hassanipour-Azgomi S, Mohammadian-Hafshejani A, Ghoncheh M, Towhidi F, Jamehshorani S, Salehiniya H (2016). Incidence and mortality of prostate cancer and their relationship with the Human Development Index worldwide. Prostate Int.

[3] Heyns CF (2008). Is prostate cancer more common and more aggressive in African men?. Afr J Urol.

[4] Epstein JI, Lotan TL (2015). The lower urinary tract and male genital system. Robbins and Cotran Pathologic Basis of Disease.

[5] Chen N, Zhou Q (2016). The evolving Gleason grading system. Chin J Cancer Res.

[6] McKenney JK (2018). Prostate and seminal vesicles. Rosai and Ackerman’s Surgical Pathology.

[7] Muñoz E, Gómez F, Paz JI, Casado I, Silva JM, Corcuera MT, Alonso MJ (2003). Ki-67 immunolabeling in pre-malignant lesions and carcinoma of the prostate. Histological correlation and prognostic evaluation. Eur J Histochem.

[8] Mucci NR, Rubin MA, Strawderman MS, Montie JE, Smith DC, Pienta KJ (2000). Expression of nuclear antigen Ki-67 in prostate cancer needle biopsy and radical prostatectomy specimens. J Natl Cancer Inst.

[9] Rajeswari K, Meenakshisundaram K, Anushuya G, Rajalakshmi V (2016). Ki67 as a prognostic marker in comparison with Gleason’s grading system in prostatic carcinoma. Indian J Pathol Oncol.

[10] Hammarsten P, Josefsson A, Thysell E (2019). Immunoreactivity for prostate specific antigen and Ki67 differentiates subgroups of prostate cancer related to outcome. Mod Pathol.

[11] Carneiro A, Kayano PP, Sowalsky AG (2018). Immunohistochemical evaluation of p53, Ki67, ERG, MYC and PTEN in Gleason pattern 3 prostate cancer: implication in active surveillance. J Clin Oncol.

[12] Kaur H, Paul M, Manjari M, Sharma S, Bhasin TS, Mannan R (2016). Ki-67 and p53 immunohistochemical expression in prostate carcinoma: an experience from a tertiary care centre of North India. Ann of Pathol and Lab Med.

[13] Missaoui N, Abdelkarim SB, Mokni M, Hmissa S (2016). Prognostic factors of prostate cancer in Tunisian men: immunohistochemical study. Asian Pac J Cancer Prev.

[14] Berney DM, Gopalan A, Kudahetti S (2009). Ki-67 and outcome in clinically localised prostate cancer: analysis of conservatively treated prostate cancer patients from the Trans-Atlantic Prostate Group study. Br J Cancer.

[15] Ogunbiyi JO, Shittu OB (1999). Increased incidence of prostate cancer in Nigerians. J Natl Med Assoc.

[16] Obiorah CC, Nwosu SO (2011). A histopathological study of carcinoma of the prostate in Port Harcourt, Nigeria. Niger J Clin Pract.

[17] Aligbe JU, Forae GD (2013). Prostatic tumours among Nigerian males: a private practice experience in Benin-City, South-South, Nigeria. Niger Postgrad Med J.

[18] Al-Nuaimy WM, Al-Allaf LI, Alnaimi HA (2011). P53 expression in prostatic cancer: an immunohistochemical study. Jordan Med J.

[19] Welén K, Damber JE (2022). Androgens, aging, and prostate health. Rev Endocr Metab Disord.

[20] Banerjee PP, Banerjee S, Brown TR, Zirkin BR (2018). Androgen action in prostate function and disease. Am J Clin Exp Urol.

[21] van Leenders GJ, van der Kwast TH, Grignon DJ (2020). The 2019 International Society of Urological Pathology (ISUP) consensus conference on grading of prostatic carcinoma. Am J Surg Pathol.

[22] Badmus TA, Adesunkanmi AR, Yusuf BM (2010). Burden of prostate cancer in southwestern Nigeria. Urology.

[23] Ajape AA, Ibrahim KO, Fakeye JA, Abiola OO (2010). An overview of cancer of the prostate diagnosis and management in Nigeria: the experience in a Nigerian tertiary hospital. Ann Afr Med.

[24] Anunobi CC, Akinde OR, Elesha SO, Daramola AO, Tijani KH, Ojewola RW (2011). Prostate diseases in Lagos, Nigeria: a histologic study with tPSA correlation. Niger Postgrad Med J.

[25] Raphael JE, Danagogo O (2021). Pilot study on the locoregional demographics of prostate cancer in River State, Nigeria. Niger Med J.

[26] Andrés-Sánchez N, Fisher D, Krasinska L (2022). Physiological functions and roles in cancer of the proliferation marker Ki-67. J Cell Sci.

[27] Richardsen E, Andersen S, Al-Saad S (2017). Evaluation of the proliferation marker Ki-67 in a large prostatectomy cohort. PLoS One.

[28] Muralidhar V, Ziehr DR, Mahal BA (2015). Association between older age and increasing Gleason score. Clin Genitourin Cancer.

[29] Kammerer-Jacquet SF, Ahmad A, Møller H (2019). Ki-67 is an independent predictor of prostate cancer death in routine needle biopsy samples: proving utility for routine assessments. Mod Pathol.

